# Bumblebees land remarkably well in red–blue greenhouse LED light conditions

**DOI:** 10.1242/bio.046730

**Published:** 2020-06-11

**Authors:** Lana J. de Vries, Frank van Langevelde, Coby van Dooremalen, Ilse G. Kornegoor, Martin J. Lankheet, Johan L. van Leeuwen, Marc Naguib, Florian T. Muijres

**Affiliations:** 1 Experimental Zoology Group, Wageningen University & Research, De Elst 1, 6708WD Wageningen, The Netherlands; 2 Wildlife Ecology and Conservation Group, Wageningen University & Research, Droevendaalsesteeg 3a, 6708PB Wageningen, The Netherlands; 3 Bees@wur, Business Unit Biointeractions & Plant Health, Wageningen University & Research, Droevendaalsesteeg 1, 6708PB Wageningen, The Netherlands; 4 Behavioural Ecology Group, Wageningen University & Research, De Elst 1, 6708WD Wageningen, The Netherlands

**Keywords:** *Bombus terrestris*, Insect flight, Insect vision, Landing behaviour, Light spectrum, Pollination

## Abstract

Red–blue emitting LEDs have recently been introduced in greenhouses to optimise plant growth. However, this spectrum may negatively affect the performance of bumblebees used for pollination, because the visual system of bumblebees is more sensitive to green light than to red–blue light. We used high-speed stereoscopic videography to three-dimensionally track and compare landing manoeuvres of *Bombus terrestris* bumblebees in red–blue light and in regular, broad-spectrum white light. In both conditions, the landing approaches were interspersed by one or several hover phases, followed by leg extension and touchdown. The time between leg extension and touchdown was 25% (0.05 s) longer in red–blue light than in white light, caused by a more tortuous flight path in red–blue light. However, the total landing duration, specified as the time between the first hover phase and touchdown, did not differ between the light conditions. This suggests that the negative effects of red–blue light on the landing manoeuvre are confined to the final phase of the landing.

This article has an associated First Person interview with the first author of the paper.

## Abstract (precis)

**Summary:** Red–blue LED lights are being used in greenhouses to improve plant growth. The changed light spectrum may affect pollinators. We studied how these lights affect landing performance of foraging bumblebees.

## INTRODUCTION

Bumblebees (*Bombus spp.*) are highly efficient pollinators. They visit more flowers, forage over a larger range and can carry higher loads of pollen on their body than honeybees (Banda and Paxton, 1991; Willmer et al., 1994). They are also relatively well adapted to cold weather and limited light conditions (Corbet et al., 1993; Reber et al., 2016a). While bumblebees depend highly on visual information during flight, they can be found foraging during twilight hours when light intensity is low (Spaethe and Weidenmüller, 2002; Kevan et al., 2009). For these reasons, bumblebees are currently the primary natural pollinator used in greenhouses (Velthuis and Doorn, 2006).

Although commercial bumblebee colonies are used on a large scale for pollination, bumblebee colonies kept in greenhouses are still doing less well than wild bumblebee colonies. Worker bumblebees of greenhouse colonies have a reduced lifespan and the colonies themselves show limited growth (Whittington and Winston, 2003; Blacquière et al., 2007). Several possible factors have been proposed that might explain these observations, among which are the relatively low environmental variation in greenhouses and the high density of neighbouring bumblebee colonies (Birmingham and Winston, 2004; Birmingham et al., 2004). Additionally, the light conditions in greenhouses may also negatively affect greenhouse colonies (Blacquière et al., 2007).

Greenhouses often use additional artificial light to increase light intensity and day length for plant growth, especially during winter. Some greenhouses manipulate the light conditions even further by also manipulating the spectral composition of the light (Hemming, 2013; Singh et al., 2015). The development of light emitting diodes (LEDs) made it possible to specify the spectrum of the emitted light with high precision. For example, the combination of 440–480 nm blue and 640–660 nm red LED lights has been tested frequently in greenhouses (e.g. Olle and Viršilė, 2013). This spectral composition is used because plants mainly use the red and blue parts of the light spectrum for photosynthesis and growth regulation (Olle and Viršilė, 2013; Singh et al., 2015). While the implementation of these differently coloured lights in greenhouses may change the environmental conditions for pollinating bumblebees, the effect on foraging behaviour of bumblebees has not yet been studied.

We expected that removing the green part of the light spectrum would decrease the flight ability of pollinating bumblebees. Bumblebees have a visual system similar to honeybees, which are most sensitive to green light (Dyer et al., 2011). Out of nine photoreceptors in each ommatidium of the honeybees’ compound eyes, six are sensitive to green light, the function of one of the remaining photoreceptors is not yet clear and the other two are sensitive to ultraviolet and blue light (Wakakuwa et al., 2005). No photoreceptors are specifically sensitive to red light. Moreover, bumblebees rely on their achromatic visual system for three-dimensional vision and for motion detection (Paulk et al., 2008). In contrast to the chromatic visual system used for colour vision, the achromatic system primarily uses the input from the green-sensitive photoreceptors (Paulk et al., 2008; Rusanen et al., 2017). Artificial light that omits the part of the spectrum for which the achromatic system is sensitive may thus specifically affect both three-dimensional and motion vision, which are highly important during flight.

Foraging bumblebees rely on their flight capabilities when searching and collecting nectar and pollen. By flying, a bumblebee can travel relatively large distances and visit flowers that are difficult to reach. As a result, during a 1 h foraging trip, a bumblebee can visit more than 1000 flowers (Heinrich, 1979; Spaethe and Weidenmüller, 2002). To approach and land on each flower, a bumblebee primarily uses its visual-motor system to accurately control its landing dynamics (Reber et al., 2016b; Baird et al., 2013). Thus, landing manoeuvres require precise input from the visual system, and therefore we expected that specifically these manoeuvres would be negatively affected by the changes towards a red–blue light spectrum as used in greenhouses. In addition, the combination of the high number of landings required for foraging and its high demand on the visual–motor system makes the landing manoeuvre a particularly relevant manoeuvre of foraging insects to be tested under these light conditions.

**Fig. 1.**
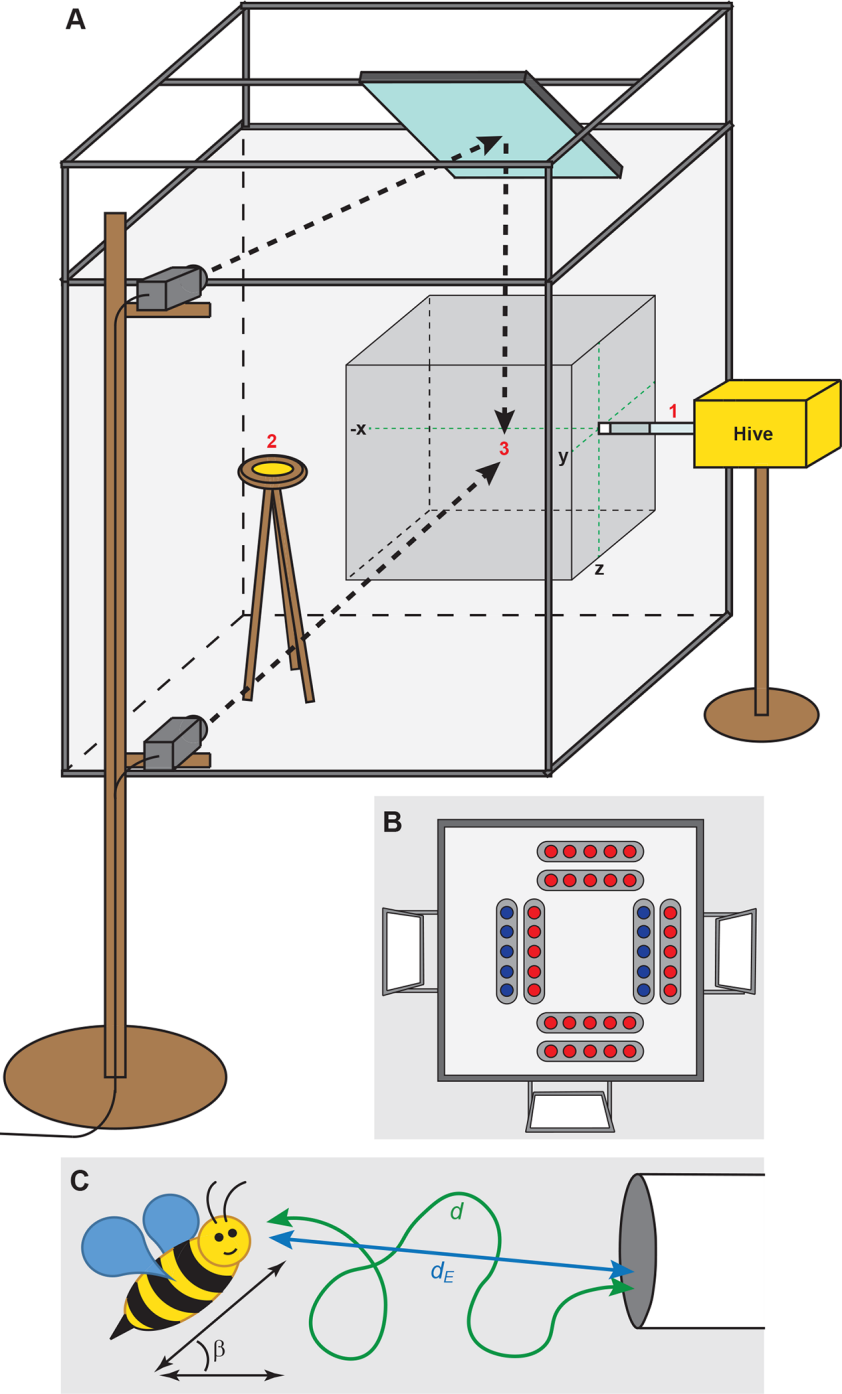
**Experimental set-up and trajectory parameters of a flying bumblebee.** (A) Overview of the experimental set-up: (1) landing tube, at 60 cm height; (2) feeding platform (5 cm diameter), positioned on a tripod at 60 cm height; (3) filming area (50×50×50 cm), filmed from the side and from above (using a silver front-surface mirror), including the x-, y- and z-axes of the coordinate system (green dotted lines). (B) Position of light sources, viewed upwards from within the set-up; the landing tube is on the right. The red and blue LED lights are laying on the top panel, while the white lights are positioned 25 cm above, at an angle of 30°. (C) A flying bumblebee including body pitch angle β, flight trajectory *d* and Euclidian distance *d_E_*.

**Fig. 2.**
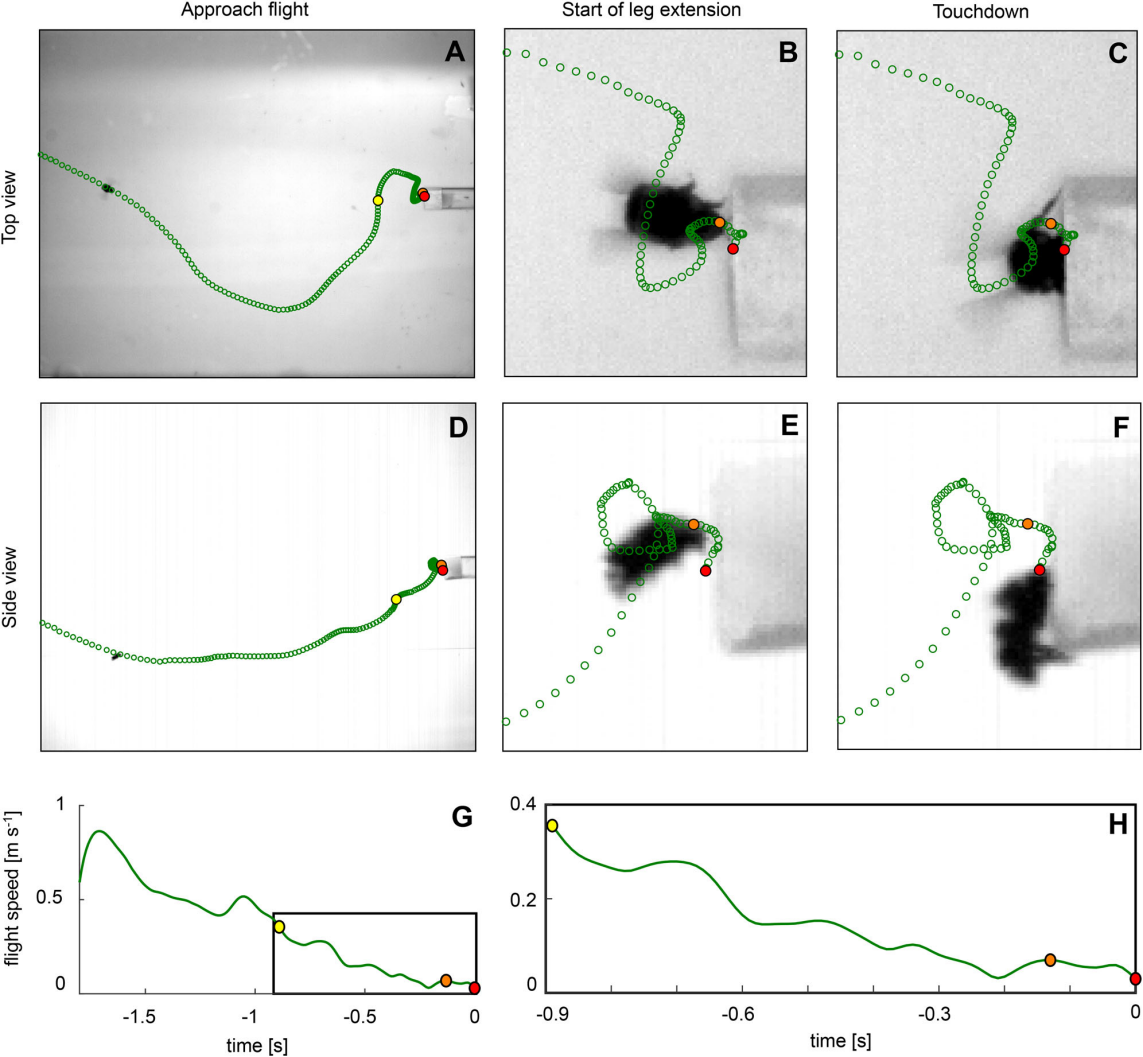
**Example landing trajectory of a bumblebee.** Top view (A–C) and side view (D–F) of a landing trajectory of a bumblebee flying in white light. Start of total landing (TL, yellow dot), start of leg extension (LE, orange dot) and touchdown (red dot). Video frames are of TL (A,D), start of LE (B,E), and touchdown (C,F). Flight speed throughout time for the complete flight (G) and from the start of TL (H).

Here, we studied how the landing performance of foraging bumblebees is affected by the red–blue greenhouse lighting system. We exposed bumblebees from a *Bombus terrestris* bumblebee colony during foraging to either red–blue light or broad-spectrum white light, and quantified their three-dimensional landing manoeuvres using stereoscopic high-speed videography tracking (Fig. 1A). We analysed the flight dynamics during two parts of the landing manoeuvre (Fig. 2): (1) the total landing (TL), which started at the first hovering manoeuvre in front of the landing platform and ended at touchdown; and (2) the leg extension phase (LE), which started at the onset of leg extension and ended at touchdown. The duration of LE was 25% longer in red–blue light, but TL duration did not significantly differ between the two light conditions. This shows that the detrimental effect of red–blue greenhouse lighting on the landing dynamics of foraging bumblebees is limited to the final landing phase.

## RESULTS

### Landing duration

**Fig. 3.**
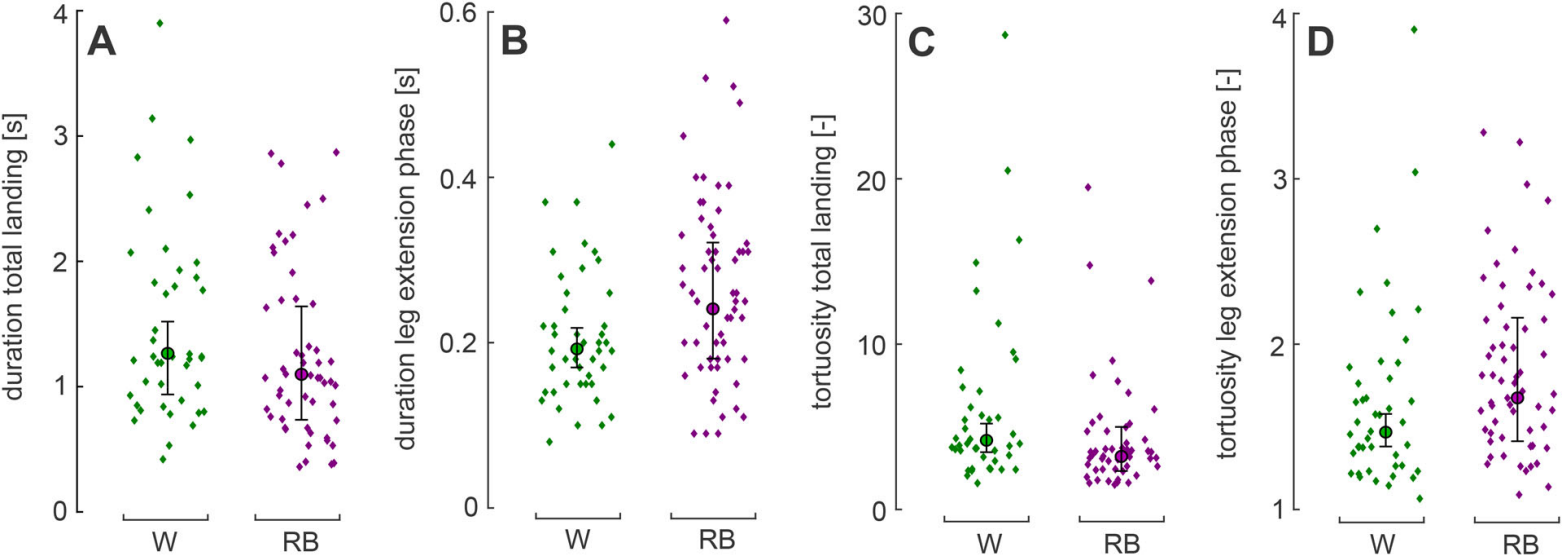
**Duration and tortuosity of landing phases.** (A) Duration of TL; (B) duration of LE; (C) tortuosity of TL; (D) tortuosity of LE. Diamonds represent analysed landing manoeuvres in white light (green) and red–blue light (purple). Circles with error bars represent back transformed means with 95% confidence intervals. Linear mixed model test results: (A) *F*_1,87_=1.67, *n*_w_=43, *n*_rb_=53, *P*=0.20; (B) *F*_1,95_=7.45, *n*_w_=44, *n*_rb_=60, *P*=0.0076; (C) *F*_1,87_=5.07, *n*_w_=43, *n*_rb_=53, *P*=0.027; (D) *F*_1,95_=6.66, *n*_w_=44, *n*_rb_=60, *P*=0.011.

To determine the landing performance of the bumblebees, we first tested whether the light conditions affected the duration of the total landing manoeuvre (Δ*t*^TL^) and the duration of LE (Δ*t*^LE^). The light conditions did not significantly affect the TL duration (back transformation of linear mixed model prediction: Δ*t*^TL^_w_=1.27 s; Δ*t*^TL^_rb_=1.10 s; *F*_1,87_=1.67; n_w_=43, *n*_rb_=53; *P*=0.20; Fig. 3A; here, w stands for white light, and rb for red–blue light; see Table 1 for nomenclature and statistical results). The duration of LE was significantly longer in red–blue light than in white light (Δ*t*^LE^_w_=0.19 s; Δ*t*^LE^_rb_=0.24 s; *F*_1,95_=7.45; n_w_=44, *n*_rb_=60; *P*=0.0076; Fig. 3B).

**Table 1.**
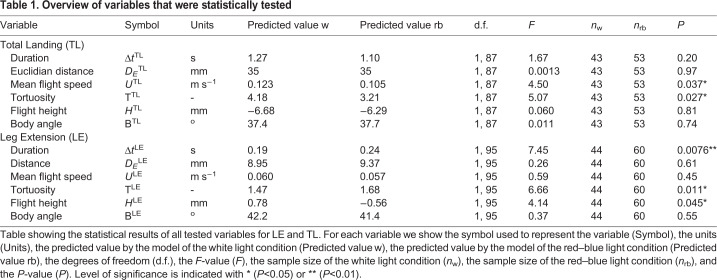
**Overview of variables that were statistically tested**

### Euclidian distance, flight speed and tortuosity

**Fig. 4.**
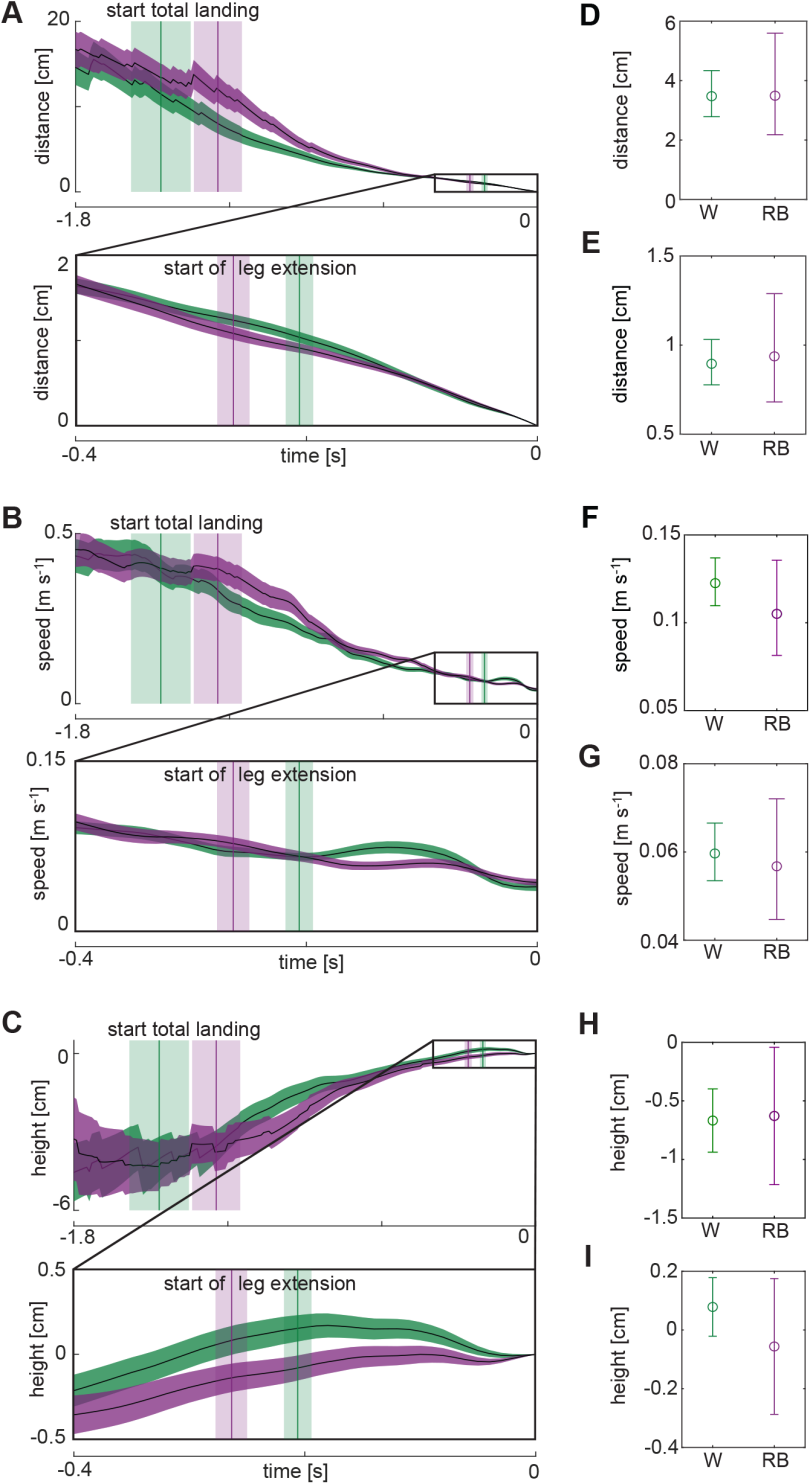
**Euclidian distance to platform, flight speed and flight height of bumblebees.** The average landing dynamics of bumblebees flying in red–blue light (purple) and in white light (green). (A–C) Temporal dynamics of the Euclidian distance to platform *d_E_* (A), flight speed |**U**| (B), and flight height *h* (C), with *t*=0 s at touchdown. Results are shown per light condition as mean (black line) and standard errors of the means (colour band). Each panel consists of two views: temporal dynamics for −1.8<*t*<0 s including the mean and standard error of the start of the total landing for both groups (vertical lines with shading); temporal dynamics for −0.4<*t*<0 s including the mean and standard error of the start of leg extension for both groups (vertical lines with shading). (D–I) Back transformed means and 95% confidence intervals for both groups of the landing dynamics metrics: Euclidean distance at the start of TL (D) and LE (E); mean flight speed during TL (F) and LE (G); mean height during TL (H) and LE (I). Linear mixed model test results: (D) *F*_1,87_=0.0013, *n*_w_=43, *n*_rb_=53, *P*=0.97; (E) *F*_1,95_=0.26, *n*_w_=44, *n*_rb_=60, *P*=0.61; (F) *F*_1,87_=4.50, *n*_w_=43, *n*_rb_=53, *P*=0.037; (G) *F*_1,95_=0.59, *n*_w_=44, *n*_rb_=60, *P*=0.45; (H) *F*_1,87_=0.060, *n*_w_=43, *n*_rb_=53, *P*=0.81; (I) *F*_1,95_=4.14, *n*_w_=44, *n*_rb_=60, *P*=0.045.

This increase in duration of LE in red–blue light could be caused by three different types of changes in flight behaviour: an increase in Euclidian distance from the bumblebee to the landing target at the start of LE (*D_E_*^LE^, Fig. 1C), a decrease in flight speed throughout LE (*U*^LE^) or an increase in the tortuosity of the flight track (T^LE^=*D*^LE^/*D_E_*^LE^, where *D*^LE^ is the length of the flight track, Fig. 1C). Both the Euclidian distance and the mean flight speed did not differ significantly between white light and red–blue light (*D_E_*^LE^_w_=8.95 mm; *D_E_*^LE^_rb_=9.37 mm; *F*_1,95_=0.26; *n*_w_=44, *n*_rb_=60; *P*=0.61; Fig. 4A,E; *U*^LE^_w_=0.060 m s^−1^; *U*^LE^_rb_=0.057 m s^−1^; *F*_1,95_=0.59; *n*_w_=44, *n*_rb_=60; *P*=0.45; Fig. 4B,G). However, in red–blue light the bumblebees showed a significantly higher tortuosity during LE than in white light (T^LE^_w_=1.47; T^LE^_rb_=1.68; *F*_1,95_=6.66; *n*_w_=44, *n*_rb_=60; *P*=0.011; Fig. 3D). This indicates that an increased tortuosity of the bumblebee’s flightpath likely contributes most to the increase in duration of LE in red–blue light.

Despite the fact that light conditions did not affect the total landing duration, we did find differences in the flight dynamics (Figs 3 and 4). Both the mean flight speed and tortuosity were significantly lower in red–blue light than in white light during TL (*U*^TL^_w_=0.123 m s^−1^; *U*^TL^_rb_=0.105 m s^−1^; *F*_1,87_=4.50; *n*_w_=43, *n*_rb_=53; *P*=0.037; Fig. 4B,*F*; T^TL^_w_=4.18; T^TL^_rb_=3.21; *F*_1,87_=5.07; *n*_w_=43, *n*_rb_=53; *P*=0.027; Fig. 3C). The reduction in flight speed in red–blue light increased TL duration, whereas the concomitant reduction in tortuosity decreased TL duration. Therefore, the combination of reduced flight speed and tortuosity in red–blue light did not affect landing duration compared to white light. The light conditions did not affect the Euclidian distance from the target at the start of the total landing manoeuvre (*D_E_*^TL^_w_=35 mm; *D*^TL^_E_rb__=35 mm; *F*_1,87_=0.0013; *n*_w_=43, *n*_rb_=53; *P*=0.97; Fig. 4A,D).

### Flight height and body angle

To better understand the landing dynamics, we also investigated the flight height *H* and body pitch angle Β during both phases. During TL, the light conditions did not significantly affect the average height of the bumblebees (*H*^TL^_w_=−6.68 mm; *H*^TL^_rb_=−6.29 mm; *F*_1,87_=0.060; *n*_w_=43, *n*_rb_=53; *P*=0.81; Fig. 4C,H) or the average body pitch angle (Β^TL^_w_=37.4°; Β^TL^_rb_=37.7°; *F*_1,87_=0.011; *n*_w_=43, *n*_rb_=53; *P*=0.74, Fig. 1C). During LE, bumblebees flying in white light approached the landing target more often from above, while bumblebees flying in red–blue light approached the landing target from below. The relative height with respect to the landing point in white light was on average *H*^LE^_w_=0.78 mm and in red–blue light it was *H*^LE^_rb_=−0.56 mm (*F*_1,95_=4.14; *n*_w_=44, *n*_rb_=60; *P*=0.045; Fig. 4C,I). No effect of light condition on average body pitch angle was found during LE (Β^LE^_w_=42.2°; Β^LE^_rb_=41.4°; *F*_1,95_=0.37; *n*_w_=44, *n*_rb_=60; *P*=0.55).

## DISCUSSION

We tested whether the landing performance of bumblebees from a *B.* *terrestris* bumblebee colony was affected by the use of red–blue greenhouse lights, by comparing the flight dynamics of bumblebees landing in red–blue light and in broad-spectrum white light. We quantified landing performance using two metrics: the duration of the complete landing manoeuvre starting at the first hovering phase (TL), and the time between leg extension and touchdown (LE). We did not find an effect of light conditions on the duration of TL, whereas the duration of LE was 25% (0.05 s) longer in red–blue light than in white light. This suggests that the negative effect of red–blue light on the landing performance of these bumblebees is confined to the final phase of the landing.

**Fig. 5.**
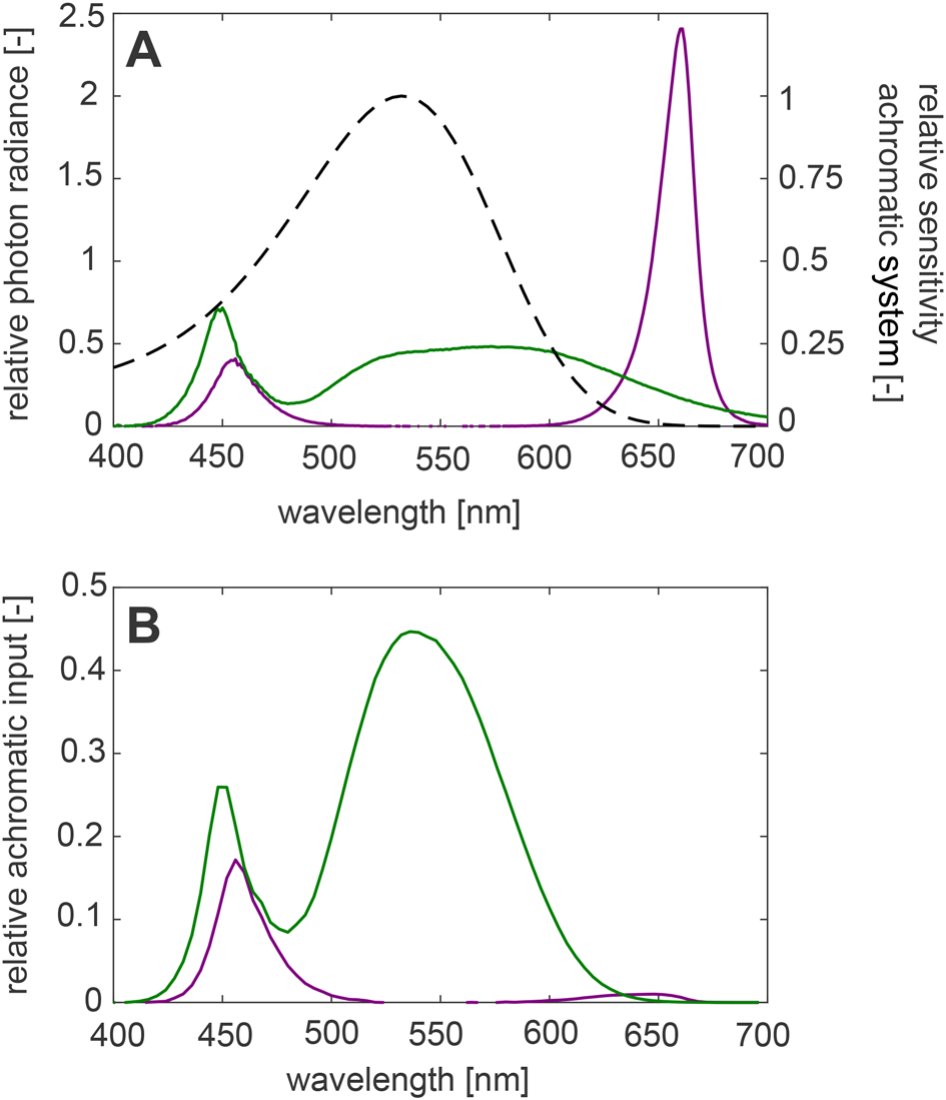
**Light spectra and bumblebee spectral sensitivity.** (A) Relative photon radiance per wavelength in the white light condition (green line, left axis) and in the red–blue light condition (purple line, left axis), as measured with a spectrometer, together with the relative sensitivity of the achromatic system of bumblebees per wavelength (dashed line, right axis; Skorupski et al., 2007). (B) Relative achromatic input in the bumblebee brain in the white light condition (green) and in the red–blue light condition (purple).

The visual system of bumblebees is most sensitive to green light, which is almost completely absent in red–blue greenhouse light. We calculated that due to this the bumblebees would perceive the red–blue greenhouse light as 8.2 times less bright than the white light (see Materials and Methods, Fig. 5). Given this significant reduction in perceived light intensity, it is striking that the landing bumblebees performed so well in red–blue greenhouse lights. How much of the increase in landing duration was due to the perceived reduction in light intensity and what aspects were due to change in colour spectrum is not known. A study in which the light spectrum is systematically changed would be needed to answer this question.

### Landing dynamics during the TL

Duration of the total landing of the bumblebees did not differ between light conditions, but we did observe a lower mean flight speed and a lower tortuosity in red–blue light than in white light during TL. So the bumblebees were flying slower in red–blue light, but they seemed to be compensating for this by flying less tortuously. This resulted in a similar duration of the TL in red–blue light as in white light. Future studies with a broader range of light conditions will be necessary to provide more insight in the mechanisms underlying this flight behaviour.

### Landing dynamics during LE

The duration of LE took longer in red–blue light than in white light, which was caused by a higher tortuosity of the flight trajectory in red–blue light. This longer duration of LE in red–blue light can be explained by the low sensitivity of bumblebees to especially red light. The input in the achromatic system of bumblebees decreases with a factor of 8.2 in the red–blue light compared with the white light conditions (Fig. 5). A longer duration of LE with decreasing light intensity was also observed by Reber et al. (2016a), who studied the effect of light intensity on the landing behaviour of bumblebees. While they did not determine the tortuosity of the flight track, a high tortuosity may also have contributed to their observed increased landing duration in low light conditions.

The increase in tortuosity and hence a longer LE, could be either due to a reduction in flight control, or result from an active behavioural response of the bumblebees to the change in light conditions. By increasing the tortuosity of its flight trajectory, a bumblebee flying in red–blue light may have increased its amount of visual information by viewing the landing target from a wider range of angles. In addition, these movements increase their ability to detect edges (Lehrer, 1996). Alternatively, the increase in tortuosity under our red–blue light conditions might have been caused by a reduction in flight stability. When light intensity decreases, the green-sensitive photoreceptors of bumblebees show a longer response time (Reber et al., 2015). This decreases the temporal resolution of their vision and increases phase lag in the visual-motor feedback loop. For flying fruit flies, such delay in the visual system causes a destabilising effect on the yaw dynamics that are involved in the control of sideways movements (Elzinga et al., 2012) and on the pitch dynamics controlling the body pitch angle (Chang and Wang, 2014). The flight stability of bumblebees flying in red–blue light might thus be similarly affected.

Next to an increase in tortuosity, a decrease in average flight height relative to the landing tube was observed in red–blue light compared to white light during LE. The bumblebees approached the landing tube from below more often in red–blue light and from above more often in white light (Fig. 4C,I). This behavioural change may be an adaptation to the light conditions, but we lack sufficient insight to give a solid explanation.

### Inside the greenhouse

Our findings suggest that *B.* *terrestris* bumblebees can still land remarkably well in red–blue greenhouse light conditions. In our study, we used a single colony, and the 105 analysed landings most likely include repeated flights of the same individuals, which we could not control for. This means that we here report on the landing dynamics of the average foraging bumblebee of this colony. Because inter-individual and inter-colony differences in bumblebee behaviour have been observed (Ings et al., 2009; Muller and Chittka, 2012), we should be careful with generalising our results. Despite this, our study is the first to investigate the effect of red–blue LED lighting on the foraging performance of bumblebees. Because the use of these light systems has increased rapidly during the last few decades (Olle and Viršilė, 2013; Singh et al., 2015), it is important to address how such red–blue LED lighting affects pollination services.

Even though we challenged the bumblebees with a relatively difficult landing platform, our flight arena setup was still a relatively simple situation compared with a greenhouse situation or the natural environment of bumblebees. For example, flight distances are larger and the presence of plants makes the environment more complex than our flight arena, and bumblebees have to land on unfamiliar and complex flower structures, instead of a familiar landing target at the hive entrance. In addition, foraging bumblebees need to complete many in-flight tasks. For example, next to performing landing manoeuvres, they need to commute and navigate from and to the hive, and search for flowers.

Flower search behaviour may be more negatively affected by changes in the light spectrum than landing behaviour. This is because bumblebees use their chromatic visual system for recognising flower colour (Hempel de Ibarra et al., 2015), and colour recognition becomes more difficult when the light spectrum is changed (Dyer and Chittka, 2004a). Bumblebees can to some extent compensate for changes in the light spectrum, since their visual processing system uses colour constancy (Chittka et al., 2014; Dyer and Chittka, 2004a). For example, it has been shown that bumblebees can quickly learn to efficiently forage in light conditions without ultraviolet light (Dyer and Chittka, 2004b). However, whether their visual system is able to compensate for the narrow-spectrum red–blue light conditions used in greenhouses has not been tested. In addition, an increase in the flower search time in red–blue light compared with white light may be expected, because bumblebees detect small flowers using their achromatic system (Skorupski et al., 2006), and the flower search time taken by bumblebees increases with decreasing light intensity (Chittka and Spaethe, 2007). Future studies tracking pollination flights in more complex spatial settings will be required to unravel additional effects of light conditions on overall performance.

Our study showed a relatively small increase in the landing duration of *B.* *terrestris* bumblebees in red–blue light conditions as used in greenhouses. As a result, consequences for the landing performance may be limited. Additional studies are needed to determine the integrative effect of greenhouse lighting on the complete foraging dynamics. We suggest that in the design of next-generation greenhouse LED lights not only will optimal plant growth have to be considered, but also their effect on pollination services.

## MATERIALS AND METHODS

### Experimental design

During the experiments, we used *B. terrestris* bumblebees bred by Koppert (Berkel en Rodenrijs, The Netherlands). The colony contained more than 50 worker bumblebees (female). The hive was connected to a flight cage (1×1×1 m) that contained a feeding platform with a 50% sugar solution (Fig. 1A). The side walls of the flight cage were made of Perspex plates, only the top plate was made of ultraviolet transmitting plastic (Plexiglas XT, UV transmitting). The sugar solution was withdrawn from a 50 ml tube that was connected to the feeding platform using a wick. A transparent Plexiglas tube (15 cm long, 2 cm diameter) connected the hive to the flight cage. The final section of the landing tube (length 1 cm) was made non-transparent white using adhesive tape. As a result, bumblebees returning to the hive would aim at this white section during their landing manoeuvre. In addition to the availability of sugar water, we fed the bumblebees inside the hive with a mixture of sugar solution and pollen twice a week, to keep the colony in good breeding condition. The temperature in the experimental room was regulated between 18–20°C.

Before the experiment, we trained the bumblebees to fly into the flight cage, land on the feeding platform, collect the sugar solution from the feeding platform, fly back to the hive and land on the final section of the landing tube. This training and all experiments were performed on the colony that remained connected to the setup throughout the study. During the day, forager bumblebees were allowed to forage *ad libitum*; at night, the exit from the hive was kept closed, to keep the bumblebees inside.

After successful training, we recorded the landings of the bumblebees on the landing tube with a high-speed stereoscopic videography system (for technical details, see Supplementary information). This videography system consisted of two synchronised cameras allowing us to reconstruct the three-dimensional flight paths of all recorded landing manoeuvres. The camera system did not allow us to identify individual bumblebees, and thus we were unable to add an ID mark to the landing manoeuvres.

We conducted the experiments within 8 days in May 2016, during which we recorded 105 stereoscopic videos of bumblebees landing on the landing tube, in the two different light conditions. These were red–blue light as often used in greenhouse plant-growth experiments (Olle and Viršilė, 2013), and a broad-spectrum white light condition as control. During most days, both light conditions were applied, one during the morning and one during the afternoon. The order of the light conditions was randomised.

### Light conditions

To precisely control the light conditions during the study, the experimental setup was placed in a completely darkened tent inside the Wageningen UR greenhouse. For the white light condition, we used three broad-spectrum white LED panels of 30×30 cm (CCT 5500–6000K, 36 watt each), powered by stabilized LED power supplies (PE298B50120, 25–42V). The three panels were attached to the aluminium framework that surrounded the flight cage (Fig. 1B). For the red–blue light condition, we used six red light LED modules (660 nm, 10 watt each) and two blue light LED modules (455 nm, 14 watt each), developed by Phillips Lighting (Eindhoven, The Netherlands). Each light module contained five small LEDs, and was powered by a dedicated stabilized LED power supply (Mean Well: PWM-40-24). We placed the light modules directly onto the UV transmitting Plexiglas (Fig. 1B). No ultraviolet light was provided.

To simulate the light conditions of the understorey inside greenhouses, we used approximately half the number of light modules as used in climate chamber experiments with tomatoes (personal communication, Y. Yi, 2019), and we diffused the light using white diffusion filters (Lee Filter 216). The spectrum of the red–blue light was similar to spectra often used in greenhouse experiments with tomato plants (Olle and Viršilė, 2013). We measured the relative wavelength spectrum of both light systems using a spectrometer (USB2000, Ocean Optics; Fig. 5A). The spectrometer measured the light spectrum reflected by a broad-spectrum reflection plate, placed inside the setup, at the height of the landing tube, and at an angle of 45° from the horizontal. By comparing the areas under the curves of the two light conditions in Fig. 5A, the photon radiance was found to be 1.35 times higher in white light than in red–blue light conditions.

We estimated the relative input of each light condition into the achromatic system of the bumblebee brain, by multiplying the wavelength spectrum of both light conditions with the sensitivity curve of the achromatic system of bumblebees (Skorupski et al., 2007) (Fig. 5A). The resulting distribution of the relative achromatic input across the light spectrum is clearly different between the red–blue and white light (Fig. 5B). By comparing the areas under these curves, the relative achromatic input into the brain was found to be 8.2 times higher in white light than in red–blue light.

### Video recordings

We used a stereoscopic high-speed videography system to track the three-dimensional flight trajectories of foraging bumblebees landing on the landing tube. The videography system consisted of two synchronised high-speed video cameras, filming at 100 frames per second (Mikrotron Eosens MC 1362; 1280×1024 pixels; top camera: 200 mm Nikkor lens; side camera: 50 mm Nikkor lens). They were synchronised using a purpose-built pulse generator. Together, the cameras provided a three-dimensional view of an approximately 50×50×50 cm area in front of the landing tube. During the experiments, the cameras were recording to a ring buffer of 500 video frames. After a bumblebee landed, the camera system was manually triggered and the last 5 s (500 frames) before the trigger point were saved and converted to lossless AVI files.

We used an infrared LED light [Bosch Aegis SuperLed, 850 nm, 10 degree beam pattern (SLED10-8BD)] as backlight illumination for each camera; bumblebees are not able to see this infrared light (Dyer et al., 2011). We used white diffusion filters (Lee Filter 216) on the bottom and sides of the flight cage to diffuse the infrared light and prevent overexposure of the cameras. As a result, we filmed the bumblebees as a shadow in front of a light background.

We calibrated the camera system both before and after the experiment using DLT (Direct Linear Transformation) in Matlab (Mathworks Inc) with the program DLTcalibration (Hedrick, 2008). For this, we placed a calibration rig with 36 randomly distributed lead beats within the stereoscopic camera view area and recorded images of this set up. Using these calibration images, we calculated the 11 DLT coefficients per camera required for three-dimensional reconstruction.

### Video analysis

The video recordings were analysed in Matlab (Mathworks Inc) using the tracking program DLTdataviewer (Hedrick, 2008). Using this program, we tracked in both camera views the head position (base of the antennae) and the tip of the abdomen of each bumblebee throughout the landing manoeuvre. This was done semi-automatically, such that when needed the tracking process could be interrupted and corrected manually. Furthermore, in each video we recorded the video frame at which the bumblebee started to extend its legs and the moment of touchdown (touching the landing tube with all legs). Fig. 2 and Movies 1 (top-view) and 2 (side-view) show an example of a tracked landing trajectory in white light, and Movies 3 (top view) and 4 (side view) show a landing in red–blue light. Using the DLT calibration, the tracking program converted the tracking results of the head and tip of abdomens into their respective three-dimensional trajectories. These trajectories were filtered using a linear Kalman smoother, which resulted in filtered three-dimensional position, velocity and acceleration vectors throughout time (*t*). The measurement-noise covariance matrix of the Kalman smoother was set to identity. For the processing noise matrix, values related to position, velocity and acceleration were set to 1, 0.1 and 0.01, respectively. The cross product of the error covariance matrices was set to zero.

We used the Kalman-filtered head position **X**(*t*) and head velocity **U**(*t*) to describe the flight dynamics throughout the landing manoeuvre. The relative positions of the head and the tip of the abdomen were used to estimate the pitch angle of the body β(*t*), defined as the angle between the long body axis (head to tip of abdomen) and the horizontal plane (Fig. 1C). To compare flight dynamics between the different landing manoeuvres, we aligned all manoeuvres in time and position relative to the moment of touchdown. Thus, at touchdown, time was zero (*t*=0 s) and the bumblebee head position was located at the centre of the world reference frame [**X**=(0,0,0) m].

Based on the position vector **X**(*t*), we determined the flight height relative to the landing point *h*(*t*)=−*z*(*t*), and the Euclidian distance between the bumblebee and its landing point, *d_E_*(*t*)=|**X**(*t*)|, throughout the complete landing manoeuvre. Based on the position vector **X**(i), we determined the length of the flight trajectory as:(1)
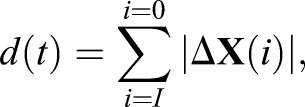
where *i* is video frame number (*i*=0 at touchdown), *I* is the video frame at time *t*, and Δ**X**(*i*) is the distance travelled at video frame *i* determined using a central difference scheme. Based on the trajectory length and Euclidian distance at each point in time, we determined the corresponding flight tortuosity τ(*t*) at that moment as (Fig. 1C):(2)
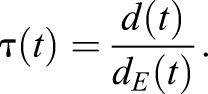


Previous studies have often observed a specific behaviour during the landing manoeuvres of bumblebees and honeybees (Evangelista et al., 2010; Baird et al., 2015; Reber et al., 2016a,b): to initiate a landing, a bee first slows down, after which it starts to slowly approach the landing platform. During this approach manoeuvre, the bee tends to perform one or multiple hovering phases, when the animal reduces its forward flight speed briefly to almost zero. Then, about 8 mm from the platform, the bee stretches its legs and performs the final touchdown manoeuvre. The time between start of leg extension and touchdown has been referred to as time-to-contact (Baird et al., 2015; Reber et al., 2016a). Based on these landing dynamics, we analysed two parts of the landing manoeuvre in our study: the TL and LE (Fig. 2). The start of TL was specified as the start of the first hovering manoeuvre in front of the platform, while the start of LE was specified as the onset of leg extension. Both landing phases ended at touchdown. LE is identical to the landing phase studied previously (Baird et al., 2015; Reber et al., 2016a).

To identify the start of the landing manoeuvre, we needed to identify the first hovering manoeuvre within each approach flight. We defined the start of the first hovering manoeuvre as the moment at which the absolute speed towards the platform reduced to below the threshold value of 0.066 m s^−1^ for the first time within the trajectory. We determined the threshold value of 0.066 m s^−1^ in three steps: (1) we calculated the speed towards the landing tube at each video frame of all trajectories by numeric time-differentiation of the Euclidian distance to the platform d_E_; (2) we made a histogram of all resulting speeds towards the landing tube, and fitted a fifth-order Gaussian model to it (Fig. S1), and (3) the threshold value was determined as the speed towards the landing tube at which the frequency was similar to the frequency at a zero speed towards landing tube (*U_P_*(*t*)=0). This was at a frequency of 392, and the corresponding speed towards the landing tube was 0.066 m s^−1^. Based on observations of the videos, we added two criteria to be certain that the bumblebee had started its landing and that it was not a coincidence that this speed threshold was reached: (1) the total speed had to be below 0.4 m s^−1^ (*U*(*t*)<0.4 m s^−1^), and (2) the Euclidian distance from the platform had to be below 15 cm (d_E_<0.15 m).

For each landing manoeuvre, we determined the duration of both TL and LE as Δ*t*^TL^=*t*^TL^ and Δ*t*^LE^=*t*^LE^, where *t*^TL^ and *t*^LE^ are the time at the start of TL and LE, respectively. Then we determined the corresponding trajectory distances for each phase, [*D*^TL^=*d*(*t*^TL^); *D*^LE^=*d*(*t*^LE^)], Euclidian distances [*D_E_*^TL^=*d_E_*(*t*^TL^); *D_E_*^LE^=*d_E_*(*t*^LE^)] and tortuosity [T^TL^= τ(*t*^TL^); T^LE^=τ(*t*^LE^)]. Within each phase, we also determined the mean flight height 
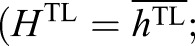
 
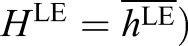
, mean flight speed 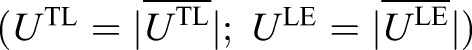, and mean body-pitch angle 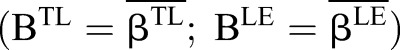. A Matlab dataset including Kalman-filtered head position, head velocity and the position of the tip of the abdomen of all flight trajectories, together with the flight variables mentioned above, is included in the Supplementary information (Dataset 1).

### Statistics

TL and LE were analysed separately. A total of 105 landings were recorded and digitally tracked, 44 in white light and 61 in red–blue light. Since we did not have an estimate of the variation of the data beforehand, we recorded a large number of flights to ensure adequate power in our statistical analyses. Trajectories for which the start of the total landing could not reliably be determined were removed from the analyses of TL. This was either because the 5 s of the recordings were too short to reliably determine the start of the first hovering manoeuvre (seven cases), or because LE preceded the speed threshold of the start of TL (one case). One trajectory was excluded from all analyses because it showed a failed landing attempt. Therefore, the total sample size was 96 for the analyses of TL, and 104 for the LE analyses. The data contained flights of different individuals with most likely repeat flights by the same individual. We did not control for individuals in the experiment.

For both the total landing and the leg extension phase, we tested for an effect of light condition on duration Δ*t*, Euclidean distance *D_E_*, tortuosity T, mean flight height *H*, mean flight speed *U*, and mean body pitch angle Β, using linear mixed models. We did the analyses in R version 3.5.0 (R Core Team, 2018), using the function lme from the package nlme (Pinheiro et al., 2018). When the residuals of the model were not normally distributed, we applied a log transformation or a double log transformation to the data if needed. The fixed factor in the models was light condition, which had two levels: white light, and red–blue light. Comparing models that included either day number (1–8), Part of day (morning, afternoon) or both as random factors indicated that only day number explained a small part of the variation in the data. Therefore, we included day number as a random intercept in the models. The bumblebees showed no improvement over time, the variation between the days was random.

## Supplementary Material

Supplementary information
