# Supplementary material for: Bumblebees land remarkably well in red–blue greenhouse LED light conditions
Source: Biol Open. 2020 Jun 11;9(6):bio046730. doi: 10.1242/bio.046730 (PMC7295593; doi:10.1242/bio.046730)
Supplement: Supplementary information [file biolopen-9-046730-s1.pdf]

Supplementary Information: Figures

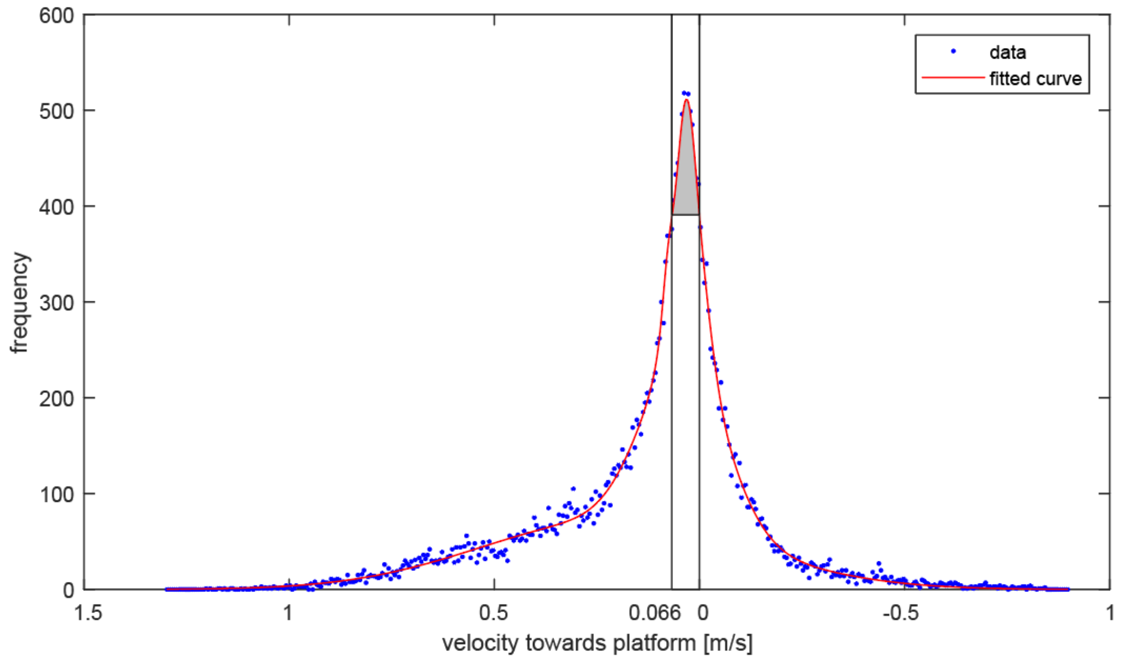

Fig. S1. Histogram of speeds towards the landing tube for all landing manoeuvres, including a fifth-order Gaussian fit. At frequency 392, the flight speed towards the landing tube was zero and 0.066 m/s. The latter value was used to identify the start of the total landing.

Biology Open • Supplementary information

Supplementary Information: Movies

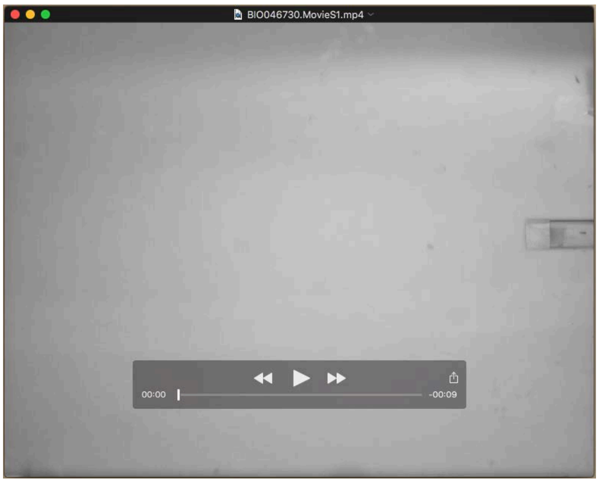

Movie 1

Landing in white light

Example of a tracked landing trajectory of a bumblebee flying in white light conditions, filmed from the top

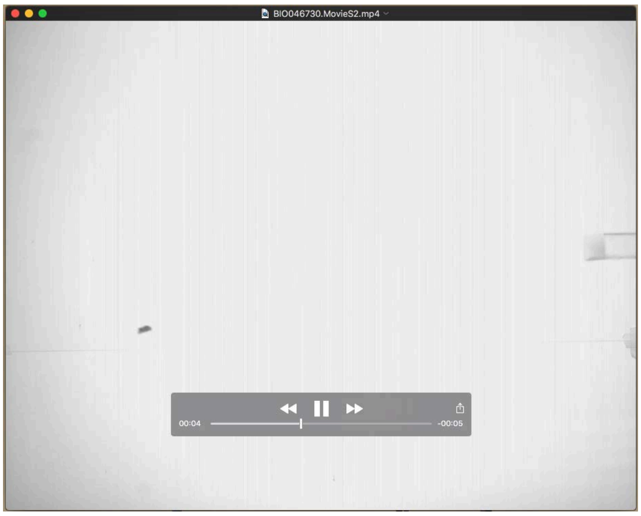

Movie 2

Landing in white light

Example of a tracked landing trajectory of a bumblebee flying in white light conditions, filmed from the side

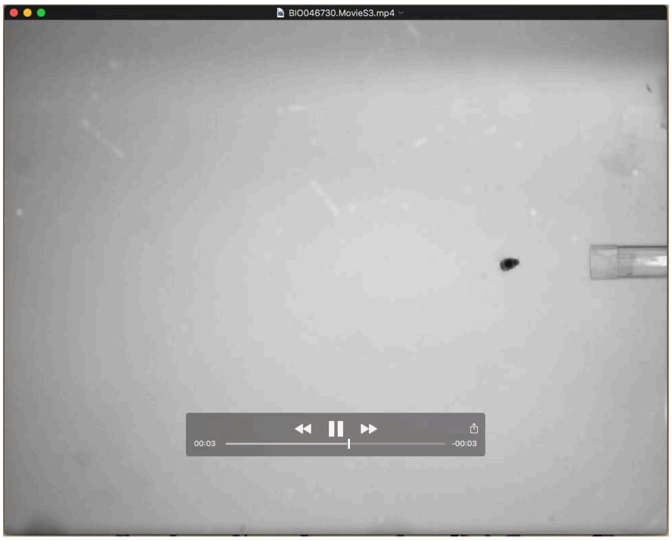

Movie 3

Landing in red-blue light

Example of a tracked landing trajectory of a bumblebee flying in red-blue light conditions, filmed from the top

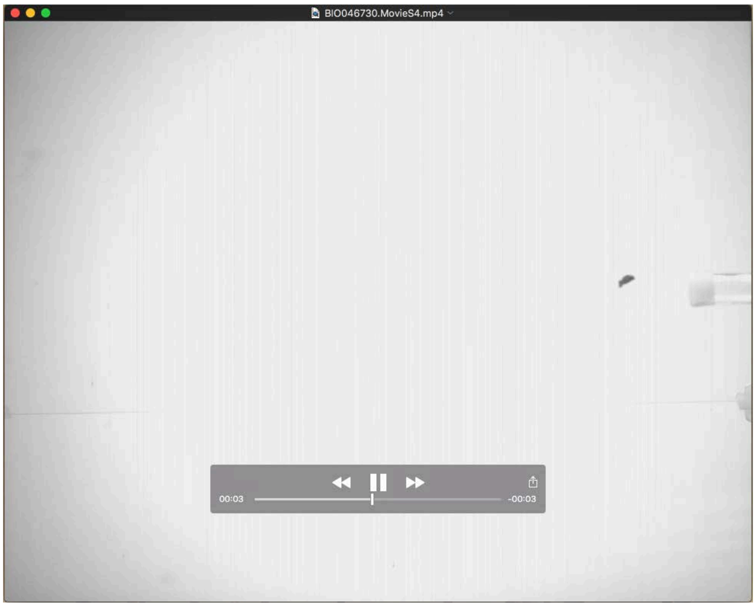

Movie 4

Landing in red-blue light

Example of a tracked landing trajectory of a bumblebee flying in red-blue light conditions, filmed from the side

Biology Open • Supplementary information

Supplementary Information: Dataset

Dataset 1. Matlab workspace including Kalman-filtered head position, head velocity**,** and the position of the tip of the abdomen of all flight trajectories, together with the flight variables, and information on treatment, date and time.

Kalman-filtered head position (mm) (position.head.x, position.head.y, position.head.z), head velocity (mm s^{-1}) (position.head.u, position.head.v, position.head.w), and position of the tip of the abdomen (mm) (position.tail.x, position.tail.y, position.tail.z) are given per time frame for all 105 flight trajectories, with frame=0 at the start of the recording, and frame=500 at touchdown.

For all flight trajectories, date of recording (info.date), time at recording (info.time) and treatment (info.treatment) are given, with 0=white light condition and 1=red-blue light condition. Info.analysed_leg_extension_phase and info.analysed_total_landing indicate whether the given flight trajectory was used during the statistical analyses of this flight phase, with 1=used and 0=not used. Timestep between consecutive frames is given by dt (s).

Variable.frame_start_total_landing and variable.frame_start_leg_extension indicate the starting frame of this flight phase for all flight trajectories, with frame=0 at the start of the recording, and frame=500 at touchdown. Variable.duration_total_landing and variable.duraiton_leg_extension_phase indicate the duration of the flight phase (s).

Variable.trajectory_distance gives the total flight trajectory distance (mm) between the position at the current frame and the position at touchdown of all 500 time frames of all 105 flight trajectories. Variable.Euclidian_distance gives the Euclidian distance (mm) between the position at the current frame and the position at leg extension. Variable.tortuosity (-) gives the tortuosity of the flight trajectory between the current position and the position at touchdown. Variable.flight_height gives the flight height (mm). Variable.flight_speed gives the flight speed (mm s^{-1}). Variable.body_pitch_angle gives the body pitch angle (°).

Click here to Download Dataset 1

Biology Open • Supplementary information
